# Sacral Herpes Zoster Mimicking Pressure Ulcers and Causing Elsberg Syndrome

**DOI:** 10.1002/ccr3.72434

**Published:** 2026-04-03

**Authors:** Junya Yamada, Akihito Yoshida, Takaaki Kobayashi, Satomi Kawamura, Sanami Doi, Tadashi Eguchi

**Affiliations:** ^1^ Department of General Internal Medicine Kameda Medical Center Kamogawa Chiba Japan; ^2^ Department of Internal Medicine University of Kentucky Lexington Kentucky USA; ^3^ Department of Dermatology Kameda Medical Center Kamogawa Chiba Japan

**Keywords:** Elsberg syndrome, herpes zoster, pressure ulcer, urinary retention

## Abstract

A 90‐year‐old woman presented with sacral skin lesions that were initially misdiagnosed as pressure ulcers. A non‐contrast abdominopelvic computed tomography revealed urinary retention, prompting further evaluation and leading to the diagnosis of sacral herpes zoster complicated by Elsberg syndrome. The patient was treated with antiviral therapy, and her urinary function gradually improved, with successful catheter removal by day 20.

## Key Clinical Message

Sacral herpes zoster may mimic pressure ulcers, particularly when lesions of varying ages are present. Careful skin examination is essential. Associated neurogenic bladder or sphincter dysfunction should raise suspicion for varicella‐zoster–associated Elsberg syndrome, in which early antiviral therapy may improve neurologic and functional outcomes.

## Graphical abstract

Close examination of the sacral lesions revealed circular lesions with post‐rupture changes (petechiae, blisters, and purpura) localized slightly to the right of the midline, mimicking a pressure ulcer. Recognition of these features, together with urinary retention and bowel dysfunction, supported the diagnosis of sacral herpes zoster complicated by Elsberg syndrome and enabled timely antiviral therapy.

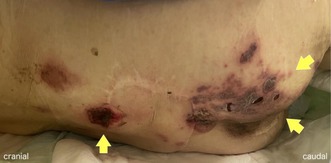

## 1 Case Presentation

**FIGURE 1:**
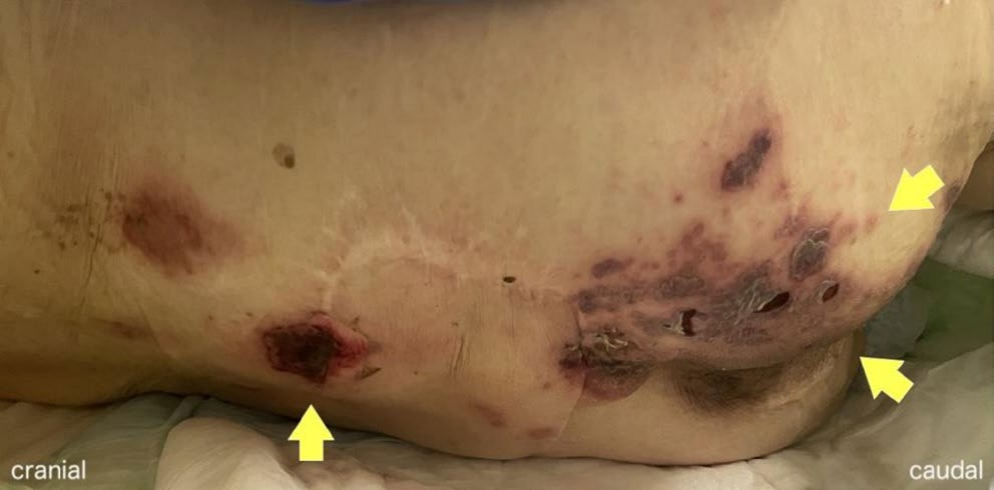
Closer examination of the sacral lesions revealed circular lesions with post‐rupture changes, including petechiae, blisters, and purpura. The rash was localized slightly to the right of the midline.

A 90‐year‐old woman residing in a nursing facility, who was ambulatory at baseline with a walker, presented following recurrent falls. Approximately 1.5 months prior to admission, she experienced a fall after which she was unable to stand from the floor and mobilized by shuffling on her buttocks. However, with staff assistance to achieve a standing position, she was subsequently able to ambulate with a walker. Two days prior to admission, she sustained another fall and again became unable to stand, even with assistance, resorting to shuffling on her buttocks for mobility. Following this event, she developed fever and fatigue and was brought for evaluation. On arrival, her temperature was 38.7°C. Physical examination revealed reduced tone of the internal anal sphincter without objective muscle weakness, sensory deficits or abnormal reflexes, as well as circumferential skin lesions in the sacral area. Owing to the patient's cognitive impairment and the absence of witnesses, reliable information regarding the timing of rash onset could not be obtained. The sacral skin lesions were initially presumed to be pressure ulcers due to their location and appearance. A non‐contrast abdominopelvic computed tomography was performed to investigate the source of fever and revealed significant urinary retention. Given the neurogenic bladder and persistent fever, closer examination of the sacral lesions was pursued. The lesions were noted to have features of vesiculation, crusting, petechiae, and purpura, distributed in a dermatomal pattern consistent with S1–S2 (first‐second sacral) nerve involvement (Figure 1). The varicella‐zoster virus (VZV) antigen swab test confirmed sacral herpes zoster. The clinical picture, accompanied by neurological signs of cystorectal dysfunction, was consistent with VZV‐induced Elsberg syndrome. The fever at admission was attributed to a urinary tract infection in the setting of urinary retention and was treated with ceftriaxone for 7 days. For urinary retention due to a neurogenic bladder secondary to Elsberg syndrome, a urinary catheter was placed and bethanechol was initiated to promote detrusor contraction. After 2 weeks without spontaneous voiding, urapidil was added to facilitate urethral sphincter relaxation. By hospital day 20, spontaneous voiding resumed, and the urinary catheter was successfully removed. The patient concurrently regained baseline ambulation with a walker, and the sacral rash resolved with residual hyperpigmentation. She was discharged after rehabilitation on hospital day 55 without recurrent urinary dysfunction.

## 2 Discussion

Elsberg Syndrome is a rare neurologic condition characterized by acute or subacute bilateral lumbosacral radiculitis or myelitis, most often triggered by viral infections. Although it has classically been associated with herpes simplex virus, varicella‐zoster virus (VZV) is also a well‐recognized cause [1]. Typical clinical manifestations reflect lumbosacral nerve root involvement [1]. Herpes zoster usually presents as a painful vesicular eruption in a dermatomal distribution; however, its clinical manifestations may vary depending on the site of viral reactivation and may include prominent neurologic involvement [2]. Sacral zoster, in particular, may be overlooked in immobile older adults as lesions often occur in pressure‐prone areas, potentially delaying appropriate management. In this case, an infected pressure injury was initially suspected given the patient's history of buttock shuffling. However, the unilateral, non–pressure‐related rash distribution, the presence of lesions at different stages, and associated cystorectal dysfunction supported a diagnosis of VZV‐induced Elsberg syndrome. Management of VZV‐related Elsberg Syndrome involves antiviral agents, symptomatic treatment, and in selected cases, corticosteroids [3]. The clinical course is variable: symptoms may resolve within days or persist for months to years, with potential for relapse. Prognosis is influenced by immune status, symptom severity, and timing of treatment initiation [1, 3]. This case highlights the importance of considering sacral herpes zoster in the differential diagnosis of presumed decubitus lesions, particularly when lesions show dermatomal distribution or mixed‐stage morphology and are accompanied by neurologic or autonomic dysfunction. Awareness of this diagnostic pitfall may facilitate earlier recognition and appropriate management, potentially improving neurologic and functional outcomes.

## Author Contributions

**Junya Yamada:** writing – original draft, writing – review and editing. **Akihito Yoshida:** writing – review and editing. **Takaaki Kobayashi:** writing – review and editing. **Satomi Kawamura:** writing – review and editing. **Sanami Doi:** writing – review and editing. **Tadashi Eguchi:** writing – review and editing.

## Funding

The authors have nothing to report.

## Ethics Statement

The authors have nothing to report.

## Consent

Written informed consent was obtained from the patient for publication of this case report and accompanying images.

## Conflicts of Interest

The authors declare no conflicts of interest.

## Data Availability

Data sharing is not applicable to this article as no data sets were generated or analyzed during the current study.

## References

[1] F. Savoldi , T. J. Kaufmann , E. P. Flanagan , M. Toledano , and B. G. Weinshenker , “Elsberg Syndrome: A Rarely Recognized Cause of Cauda Equina Syndrome and Lower Thoracic Myelitis,” Neurology Neuroimmunology & Neuroinflammation 4, no. 4 (2017): e355. 28534040 10.1212/NXI.0000000000000355 PMC5427668

[2] D. H. Gilden , B. K. Kleinschmidt‐DeMasters , J. J. LaGuardia , R. Mahalingam , and R. J. Cohrs , “Neurologic Complications of the Reactivation of Varicella‐Zoster Virus,” New England Journal of Medicine 342, no. 9 (2000): 635–645. 10699164 10.1056/NEJM200003023420906

[3] H. M. Qadri , S. Pervaiz , M. Ijaz , et al., “Elsberg Syndrome—A Systematic Review of Existing Scientific Literature From 2000–2023,” Pakistan Journal of Medical Sciences 40, no. 12(PINS) (2024): S103–S113. 10.12669/pjms.40.12(PINS).11105 PMC11654645 39703979

